# Sustaining trachoma elimination: lessons from North Africa and the Middle East

**Published:** 2019-05-13

**Authors:** Jaouad Hammou, Farzad Mohammadi, Abdulatif Al Raisi, Saleh Al Harbi

**Affiliations:** 1 PhD student: Faculty of Medicine and Pharmacy of Rabat, Mohammed V University, Rabat, Morocco.; 2 National Coordinator for Eye Health and Prevention of Blindness and Trachoma Control: Ministry of Health and Medical Education, Islamic Republic of Iran.; 3 Senior Consultant Ophthalmologist: Chairman Eye Health Strengthening, Ministry of Health, Oman.; 4 National Coordinator: Eye and Ear Health Care Program, Ministry of Health, Oman.

**Governments play a leading role in successful trachoma elimination programmes.**

**Figure:**
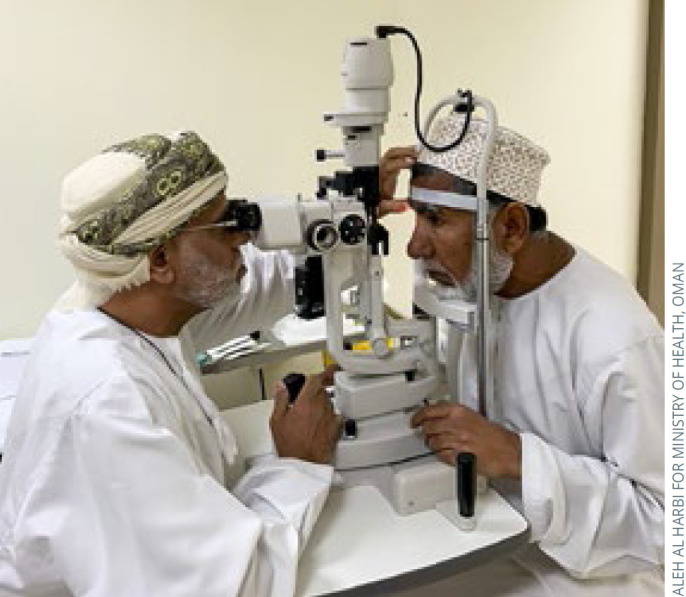
Screening for trachomatous trichiasis. OMAN

There has been significant progress to eliminate trachoma as a public health problem in the Eastern Mediterranean region. In 2012, Oman became the world's first country to be validated by the World Health Organization as having eliminated the disease, followed by Morocco in 2016 and the Islamic Republic of Iran in 2018.^1^

Government leadership, and a commitment to protect the 10.4 million people at risk of trachoma in the region have led to more than 11 million doses of antibiotics being distributed there since 2001. Access to clean water and sanitation has also improved, contributing to a reduction in transmission. However, as more countries move towards elimination targets, new strategies are needed so that national eye health care services are able to monitor, track and manage any new cases that occur and thereby prevent a resurgence of trachoma. Recent experience from Oman, Morocco and Iran provide valuable lessons.

## Morocco

In 2005, Morocco developed a robust system of epidemiological surveillance to monitor its five formerly endemic provinces. Surveillance included screening in schools, exhaustive house-to-house screening for trachomatous trichiasis (TT) and population-based surveys that included the country's most disadvantaged populations. The system was integrated into existing national epidemiological surveillance in order to improve sustainability and reduce costs.

Trachoma is continuously monitored. At the community level, school health nurses screen all primary school children through its national school health programme. Nurses at local health facilities are trained to identify and treat trachoma whenever it appears. If trachoma is detected, an investigation is conducted that includes screening of the affected person's family and close contacts. Adults are screened for TT as part of routine primary health care visits.

Access to primary health care in Morocco has been extended to 100% of the population. By integrating trachoma activities into national eye health plans, Morocco has ensured that cases are identified and managed before the disease can spread.

## Oman

Oman's work to sustain elimination demonstrates the importance of integrating surveillance into existing programmes. Most significantly, Oman integrated trachoma surveillance into its routine health services and its national school programme. Doctors at local health centres are trained to identify trachoma and school health nurses are trained to refer suspected cases to the nearest ophthalmologist. When trachoma is identified, the following steps are taken as per ministry of health guidelines:

1. Trachoma screening is conducted for all people who live with the patient.
2. Antibiotics are provided to clear infection.
3. Health and ocular hygiene education are provided.
4. The governorate's (region's) epidemiologist documents each case via the electronic Al-Shifa 3+ case monitoring system.

## Islamic Republic of Iran

Iran achieved trachoma elimination without a stand-alone programme. Trachoma was eliminated through comprehensive primary health care services and broader development objectives, such as universal access to clean water and sanitation. The steering committee for the country's national water programme is committed to providing water, sanitation and hygiene (WASH) services and continued education about facial cleanliness to areas where poor environmental sanitation remains.

The Islamic Republic of Iran demonstrates that, with strong health systems and social improvements, trachoma can be eliminated without relying on donated medicines. Iran placed a strong emphasis on establishing and strengthening its health care network, which now reaches nearly all rural areas covered by the national primary health care system.

## Conclusion

Post-elimination activities in Oman, Morocco and Iran showcase the essential element of national government leadership in their successful programmes and the importance of integrating programmes into existing systems for monitoring the health of the nation.

All national trachoma programmes and their partners can learn from these positive examples of government leadership in order to integrate ongoing surveillance and response into national eye health plans and to include elimination efforts within the WASH sector and school health programmes. This will be crucial in order to sustain the elimination of trachoma as a public health problem.
